# Near-infrared spectroscopy (NIRS) *in vivo* assessment of skeletal muscle oxidative capacity: a comparison of results from short *versus* long exercise protocols and reproducibility in non-athletic adults

**DOI:** 10.3389/fphys.2024.1429673

**Published:** 2024-07-23

**Authors:** Fistra J. Tandirerung, Alexandra Jamieson, Elizabeth Hendrick, Alun D. Hughes, Siana Jones

**Affiliations:** ^1^ MRC Unit for Lifelong Health and Ageing at UCL, Department of Population Science and Experimental Medicine, Institute for Cardiovascular Science, University College London, London, United Kingdom; ^2^ Department of Cardiology and Vascular Medicine, Sardjito Central Public Hospital, Gadjah Mada University, Yogyakarta, Indonesia

**Keywords:** near-infrared spectroscopy, skeletal muscle, oxidative capacity, exercise near-infrared spectroscopy, exercise

## Abstract

**Background:**

Near-infrared spectroscopy (NIRS) provides a non-invasive, cost-effective method for assessing skeletal muscle oxidative capacity when combined with a short exercise protocol and arterial occlusions. However, the impact of different exercise protocols and reproducibility of the method in non-athletic adults have not previously been assessed.

**Methods:**

Young, non-athletic adults (YA) were invited to perform a short duration, fast frequency contraction (SF) exercise protocol and a long duration slow frequency (LS) contraction protocol, combined with NIRS measurements and arterial occlusions to assess skeletal muscle oxidative capacity. YA and older non-athletic adults (OA; >65 years old) were invited to perform the SF exercise protocol twice to assess the reproducibility of this oxidative capacity measurement.

**Results:**

We included 25 participants (14 male (56%), age range: 18–86 years) in the analyses. There was a strong positive correlation and good agreement between time constants derived following the SF and LS exercise protocols (Lin’s concordance correlation coefficient: 0.69, *p*-value < 0.001 mean bias [LoA]: −3.2 [−31.0, 24.4] seconds. There was a strong positive correlation and good agreement between time constants derived from the SF exercise protocol in the YA & OA group (Lin’s concordance correlation coefficient: 0.63, *p*-value < 0.001; mean bias [LoA] −6.4 [−34.0, 21.3] seconds).

**Conclusion:**

These data provide evidence to suggest that NIRS is a reliable *in vivo* method for the assessment of skeletal muscle oxidative capacity irrespective of exercise protocol duration or muscle contraction frequency. NIRS-measured oxidative capacity via the SF exercise protocol was reproducible in non-athletic adults with a wide range in age.

## Introduction

Skeletal muscle function declines with age and in a multitude of disease phenotypes potentially leading to reduced physical function, frailty and loss of independence (Marzetti and Leeuwenburgh, 2006; Gomes et al., 2017). Tracking pathophysiological alterations in skeletal muscle function is critical for understanding disease mechanisms, progression and response to intervention (Coen et al., 2019). Skeletal muscle oxidative capacity is a key feature, representing the overall performance of the muscle tissue to extract and utilize oxygen, which is known to decline with aging and in the presence of many diseases (Santanasto et al., 2016; Gonzalez-Freire et al., 2018; Trevino et al., 2019). There is an urgent need to develop cost-effective, non-invasive methods for assessing oxidative capacity in skeletal muscle.

Current methods for assessing skeletal muscle oxidative capacity include tissue biopsy for high resolution respirometry (HRR) and phosphorus magnetic resonance spectroscopy (^31^P-MRS) to capture Phosphocreatine (PCr) depletion and recovery and maximal rates of ATP production via oxidative phosphorylation (Lanza et al., 2010). Although HRR provides detailed mechanistic insight into mitochondrial bioenergetic pathways, the invasive nature of the biopsy can be uncomfortable for participants and requires clinical facilities and expertise. ^31^P-MRS is the reference-standard for directly measuring maximal rates of PCr recovery following a short bout of exercise, however, is limited to specialist centres that have access to expensive scanners.

Near-infrared spectroscopy (NIRS) offers an alternative, non-invasive and cost-effective technique for estimating skeletal muscle oxidative capacity *in vivo*. When combined with low intensity exercise and subsequent transient arterial occlusions, the recovery kinetics of local muscle oxygen consumption post-exercise provide an estimate of PCr pay-back (Ryan et al., 2012; Scheeren et al., 2012; Sumner et al., 2020). Despite cross-validation of NIRS with ^31^P-MRS demonstrating a strong correlation (Nagasawa et al., 2003; Ryan et al., 2013a; Ryan et al., 2014a; Jones et al., 2016), only limited work exists investigating the exercise protocols that can be applied when using NIRS (Ryan et al., 2013b). Prior ^31^P-MRS studies have applied exercise protocols of varying lengths and frequency of muscle contractions. McCully et al utilised a 5-minute exercise phase with plantar flexion muscle contractions every 4–5 s, whereas Sedivy et al used a 6-min exercise phase with plantar flexion every 2-s (McCully et al., 1993; McCully et al., 1994; Šedivý et al., 2015). In contrast, NIRS exercise protocols described in the literature are relatively short (10–30 s) and involve rapid, vigorous contractions (Ryan et al., 2012; Ryan et al., 2013a; Ryan et al., 2013b; Ryan et al., 2014b; Sumner et al., 2020; Menon et al., 2021). A comparison of recovery time constants derived following each of these approaches has not previously been described. A 10-second short-fast protocol was chosen as it is routinely used in NIRS exercise protocol studies and is a practical approach for the future application of this method in a clinical context. It is compared with the 5-minute long-slow protocol that is generally used for ^31^P-MRS studies as the standard for *in-vivo* direct measurement of PCr recovery rates. Furthermore, assessing the reproducibility of this method in non-athletic adults and older adults, where oxidative performance is an extremely useful health metric, would be a beneficial addition to the literature. The recruitment of adults of a wide age range would also contribute to the development of this method and its application to large population-based studies.

Thus, the objectives of this study were two-fold, (1) to compare NIRS-measured skeletal muscle oxidative capacity using two different exercise protocols, a short duration rapid muscle contraction protocol (short-fast) and long duration dispersed/controlled muscle contraction protocol (long-slow), in young healthy subjects and (2) to assess the reproducibility of NIRS-measured oxidative capacity in a non-athletic adult population with a wide age range.

## Materials and methods

### Study participants

Participants were either young, healthy adults (YA) recruited from the University College London student and staff pool and invited to attend a single research visit, or were older adults (OA), >65 years old, that had previously been enrolled in a longitudinal cohort study (the Southall and Brent Revisited, SABRE study) and were undergoing an oxidative capacity measure as part of a follow-up visit (Jones et al., 2020). All procedures were in accordance with the principles of the Helsinki declaration and all participants gave written informed consent. The study procedures were approved by the reviewer board of the UCL Research Ethics Committee (21787.001) for the YA and by the National Research Ethics Service (NRES) Committee London—North Fulham for the OA SABRE study. Participants were asked not to smoke, consume alcohol, or do moderate-to-vigorous physical activity in the 24 h prior to the testing.

### Participant characteristics and anthropometrics

Year of birth, sex, and ethnicity were reported by the participant. Height was measured barefoot using a stadiometer (Seca 217; Seca, Hamburg, Germany) to the closest centimetre and weight was measured in kilograms using digital bio-impedance scales (BC-418; Tanita, IL, United States), to calculate body mass index (BMI).

### Skeletal muscle NIRS measurements

#### Device and device placement

**FIGURE 1:**
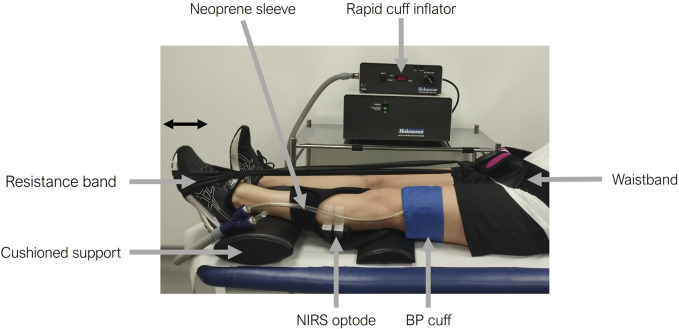
Experimental set-up for the skeletal muscle near-infrared spectroscopy (NIRS) measurements. The participant’s leg is supported with cushioned pads under the knee and ankle. A NIRS optode is positioned on the skin overlaying the gastrocnemius of the left calf, secured with micropore tape, and covered by a black neoprene sleeve. A rapidly inflatable cuff is placed above the knee (proximal to the NIRS optode) and is connected to a rapid cuff inflator. A resistance band is then placed around the participant’s foot and secured using a waistband. The participant’s foot is shown pushing against the resistance band in the plantar flexion position.

A portable continuous wave (CW) NIRS device (Portamon, Artinis Medical System, Netherlands) that measures oxy-haemoglobin (O_2_Hb) and deoxy-haemoglobin (HHb) changes at a sampling frequency of 10 Hz was used for all tests. Participants were invited to recline in a semi-supine position on a medical examination couch to reduce hemodynamic variability and optimize cardiovascular exercise adaptation (Kubota et al., 2015; Kubota et al., 2017). The small, wireless CW NIRS device was placed on the skin overlaying the left gastrocnemius muscle, held in position by micropore tape and covered with a neoprene sleeve to avoid ambient light contamination (Figure 1). The neoprene sleeve was included as part of the Artinis Medical System Portamon NIRS equipment kit and used in line with manufacturer guidelines. The leg was supported by cushioned pads placed beneath the knee and ankle. A rapidly inflatable cuff was wrapped around the leg proximal to the NIRS device above the knee and connected to a rapid cuff inflation system (Hokanson Companies, United States). A resistance band was secured around the ball of the left foot and the opposite end secured to a Velcro waistband to avoid arm fatigue during the exercise protocols or changes in tension throughout the exercise (Figure 1). The length of the resistance band was adjusted by the technician so that there was light tension in the dorsi-flexed foot position to account for differences in participant leg length.

Participants were permitted to push against the resistance band to familiarize themselves with the exercise action (plantar flexion) before resting in the baseline position. Baseline NIRS signals were acquired for at least a minute, or until stable. A resting arterial occlusion was performed for 30 s using a cuff pressure of at least 275 mmHg followed by recovery until NIRS signals returned to baseline. During the cuff inflation, visual inspection of the NIRS traces was performed to check for loss of pulsatility and reciprocity in the oxy-Hb and deoxy-Hb signals. If a complete arterial occlusion was not achieved, participants were excluded from subsequent analyses. Participants were also asked if they could tolerate the cuff inflation at the given pressure (275 mmHg).

#### Exercise protocols

##### Comparison of NIRS measured skeletal muscle oxidative capacity exercise protocols

Each participant was invited to complete two exercise protocols in series, the order of which was randomised by alternate allocation. Firstly, a “short-fast” protocol in which participants were asked to perform rapid plantar flexion against the resistance band as many times as possible for a period of 10-s and secondly, a “long-slow” exercise protocol in which participants were asked to perform 5 min of plantar flexion against a resistance band at a rate of 30 plantar flexions a minute in response to a metronome. Following each exercise protocol, short transient arterial occlusions lasting 5–8 s were applied over 3 min (5 s in the first minute and 8 s in the second and third minutes) to track recovery musVO_2_ and measure the recovery time constant (τ), an estimate of oxidative capacity (Ryan et al., 2013c; Ryan et al., 2014b; Southern et al., 2014). Longer values of τ represent poorer skeletal muscle oxidative capacity (Ryan et al., 2013c). An additional 2 min recovery period was given between each protocol.

Flow-charts illustrating the exercise protocols are presented in the supplementary information file (Supplementary Figures S1, S2).

##### Reproducibility of NIRS measured skeletal muscle oxygen consumption and oxidative capacity

After stabilisation of the trace, two resting arterial occlusions were imposed by rapidly inflating the cuff to at least 275 mmHg for 30 s on each occasion (ensuring stabilisation of the trace in between occlusions with an interval of approximately 30 s). Local skeletal muscle oxygen consumption (musVO_2_) was estimated from the change in oxy- and deoxy-Hb (Van Beekvelt et al., 2001). Each participant was invited to complete two short-fast exercise protocols in series in which participants were asked to perform rapid plantar flexion against the resistance band as many times as possible for a period of 10-s. Following each exercise protocol, short transient arterial occlusions were applied over 3 min as described above. An additional 2 min recovery period was given between each protocol.

### Adipose tissue thickness

Adipose tissue thickness (ATT) was measured at the NIRS measurement site using B-mode ultrasound (Philips EPIQ 7G Ultrasound System, Netherlands) by four experienced technicians who were involved in data capture. Three ATT measurements were recorded for each participant and averaged.

### Data processing

NIRS data were processed using custom written scripts in MATLAB R2021a (The MathWorks, United States) (Ryan et al., 2012). All processing was performed by one experienced technician. For each participant, all traces were plotted and assessed by eye for the presence of artifacts or incomplete arterial occlusion. Incomplete arterial occlusions were identified by pulsatility in the NIRS signals, a lack of reciprocity of the oxy-Hb and deoxy-Hb signals, or noise in the signal whereby the downward slope of the oxy-Hb signal could not be visualised during cuff inflation. The start of the resting arterial occlusion and each intermittent arterial occlusion was selected by eye, all further processing was automated.

Muscle oxygen consumption (musVO_2_) was measured as the downward slope of the O_2_Hb to HHb difference signal during each occlusion using at least 40 data points (4 s). The difference signal was used to account for potential shifts in blood volume into the region of interrogation during the cuff inflation (Ryan et al., 2012). The time constant (τ) was calculated using the repeated post-exercise musVo_2_ measurements from the transient occlusions. These were fit to a mono-exponential curve, visually inspected and only curves with a good fit (r^2^ > 0.70) were included in subsequent analyses. A sensitivity analysis was performed using thresholds of r^2^ > 0.60 and r^2^ > 0.65, however, the pattern of results remained similar. The time constant for recovery indicating oxidative capacity was derived from the fit as described previously (Ryan et al., 2014b).

### Statistical analysis

Statistical analysis was performed with STATA MP17 (StataCorp, United States). Categorical descriptive data are presented as n (%) and continuous variables are presented as mean ± standard deviation (SD) if normally distributed or as median [interquartile range, IQR] if skewed. The Shapiro-Wilk test was used to formally test the assumption of data normality and data were visualised with histograms. Correlations were assessed using Pearson`s or Spearman`s correlation coefficient, depending on data normality. Lin’s concordance correlation coefficients (CCC) and Bland-Altman plots [presented as mean bias (Limits of Agreement; LoA)] were used to assess the level of agreement between measurements derived from the short-fast and long-slow exercise protocols and between measurements derived from test 1 *versus* test 2 of the short-fast exercise protocol in the reproducibility study. For resting and end-exercise musVO_2_ assessment, the negative slope values were converted to positive values (multiplying by −1) to simplify the analysis and interpretation. The level of significance was set at *p* < 0.05.

## Results

In total, 16 YA were recruited for the exercise protocol comparison study. In 3 YA participants, time constants for the recovery of mus $\dot{V}$ O_2_ were excluded from summary analyses due to mono-exponential fits not exceeding our quality inclusion criteria (r^2^ > 0.70). We believe this is due to a failure of the muscle tissue to fully recover following the exercise and discuss potential explanations in more detail in the discussion section. We excluded one additional YA participant due to an observed positive change in O_2_-Hb during the resting occlusion, indicating that an arterial occlusion had not been achieved; potential explanations for this are also described in the discussion section. Therefore, 13 of the 16 YA participants recruited were included in the short-fast vs. long-slow resting and end exercise musVO_2_ analysis, and 12 participants were included in the time constant analysis, respectively.

Twenty eight participants (14 YA and 14 OA) were recruited for the reproducibility study. In three participants, time constants for the recovery of musVO_2_ were excluded from summary analyses due to mono-exponential fits not exceeding our quality inclusion criteria (r^2^ > 0.70). We excluded two additional participants from the reproducibility of resting arterial occlusion assessment due to either an observed positive change in O_2_-Hb during the resting occlusion, indicating that an arterial occlusion had not been achieved. Therefore, 25 of the 28 participants recruited were included in the time constant reproducibility analysis and 23 participants were included in the resting musVO_2_ reproducibility analysis, respectively.

### Participant characteristics

13 YA (9 male (70%), age 26 ± 3 years old) were included in the summary analyses for the exercise protocol comparison and 25 adult participants [14 male (56%), age 59 (26, 78) years old] were included in the summary analyses for the reproducibility study. YA participants in the exercise protocol comparison study were predominantly of self-reported Asian ethnicity (77%). By study design SABRE participants (OA included in the reproducibility analyses) were either first generation Indian Asian, African Caribbean or of White European ethnicity. This was because one of the primary research questions of the original SABRE study addresses health disparities by ethnic group in the UK (Jones et al., 2020). ATT was <1.5 cm in all participants. A summary of participant characteristics is presented in Table 1.

**TABLE 1:** Participant characteristics for 13 young adults included in the short-fast (SF) *versus* long-slow (LS) exercise protocol comparison and 25 adults included in the SF reproducibility study.

Mean ± SD, median [IQR] or n (%)		
	SF vs. LS exercise (*n* = 13)	SF reproducibility (*n* = 25)
Age (years)	26 ± 3	59 [26, 78]
Sex, male	9 (70%)	14 (56%)
Ethnicity		
White european	1 (8%)	17 (61%)
Asian	10 (77%)	7 (25%)
African caribbean	2 (15%)	4 (14%)
Height (cm)	172.2 ± 12.1	1,697 ± 8.6
Weight (kg)	73.2 ± 16.9	71.1 ± 16.3
BMI (kg/m^2^)	23.5 [21.5, 26.5]	22.8 [21.7, 25.4]
ATT (cm)	0.57 ± 0.29	0.51 ± 0.22

ATT, Adipose Tissue Thickness; BMI, Body Mass Index.

### Short-fast *versus* long-slow exercise protocol

**FIGURE 2:**
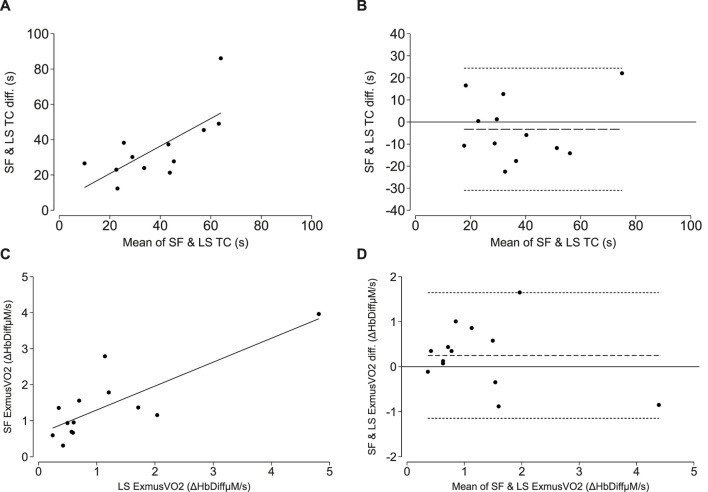
NIRS-measured oxidative capacity and end exercise muscle oxygen consumption (ExmusVO2) measure from short-fast (SF) and long-slow (LS) exercise protocols. **(A, C)** correlations between the SF and LS exercise protocol time constant (TC; τ) and SF and LS end exercise oxygen consumption. The line of best fit is plotted in solid black. **(B, D)** Bland-Altman plots demonstrating levels of agreement between the SF and LS time constant (TC; τ) and SF and LS end exercise oxygen consumption. The mean difference is plotted as the long-dashed line and the upper and lower limits of agreement are plotted as short-dashed lines.

Representative time constant curves generated from the short-fast and long-slow exercise protocols for a YA participant are included in the supplementary information file (Supplementary Figures S3, S4). The mean time constants (τ) derived after the short-fast and long-slow exercise protocols for 12 participants were 35.1 ± 19.2 s and 38.4 ± 17.3 s, respectively. There was a strong positive correlation between the two measurements (r = 0.70, *p*-value = 0.011; Lin’s CCC = 0.69, *p*-value <0.001; Figure 2A). The bias was small without indication of systematic bias, but the limits of agreement were fairly wide [mean bias (LoA): −3.2 (−31.0, 24.4) seconds; Figure 2B].

#### Resting and end-exercise musVO_2_

The mean resting musVO_2_ for 13 participants was 0.12 ± 0.09 Hb_Diff_µM/s. The mean end-exercise musVO_2_ derived after the short-fast and long-slow exercise protocols were 1.39 ± 1.00 and 1.44 ± 1.23 Hb_Diff_µM/s, respectively. There was a strong positive correlation between end-exercise musVO_2_ measured during the short-fast *versus* long-slow exercise protocols (r = 0.82, *p*-value <0.001; Lin’s CCC = 0.78, *p*-value <0.001; Figure 2C). A Bland-Altman plot depicts the mean bias and limits of agreement [Mean bias (LoA): 0.25 (−1.15, 1.65) Hb_Diff_µM/s; Figure 2D].

### Reproducibility study

**FIGURE 3:**
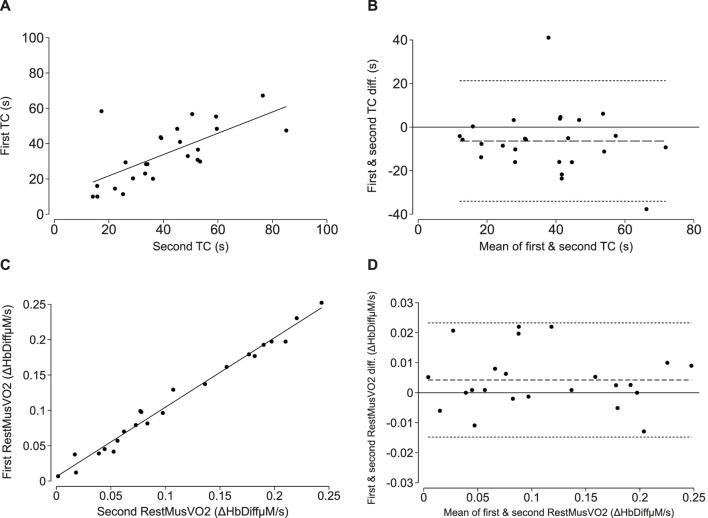
Reproducibility of NIRS-measured oxidative capacity and resting muscle oxygen consumption (RestmusVO2) in adults. **(A, C)** correlations between the first and second time constant (TC; τ) and first and second resting oxygen consumption measure. The line of best fit is plotted in solid black. **(B, D)** Bland-Altman plots demonstrating levels of agreement between the first and second time constant (TC; τ) and first and second resting oxygen consumption measure. The mean difference is plotted as the long-dashed line and the upper and lower limits of agreement are plotted as short, dashed lines.

The mean values for the first and second time constant derived from the short-fast exercise protocol were 34.0 ± 16.4 s and 40.4 ± 18.5 s, respectively. There was a strong positive correlation (cc = 0.68, *p* < 0.001; Lin’s CCC = 0.63; *p* < 0.001; Figure 3A) and good agreement between the first and second measurements with a mean difference [LoA] of −6.4 [−34.0, 21.3] seconds (Figure 3B).

#### Resting musVO_2_

The mean values for the first and second resting musVO_2_ measurements were 0.11 ± 0.07 Hb_Diff_µM/s and 0.11 ± 0.07 Hb_Diff_µM/s, respectively. There was a positive correlation (cc = 0.99, *p* < 0.001; Lin’s CCC = 0.99, *p* < 0.001; Figure 3C) and good agreement between the first and second musVO_2_ measurements with a mean difference [LoA] of 0.004 [−0.02, 0.02] Hb_Diff_µM/s (Figure 3D).

## Discussion

In this study we present evidence for good agreement between NIRS measures of oxidative capacity (τ) in the gastrocnemius performed using a short, rapid-contraction (short-fast) *versus* long, low frequency contraction (long-slow) exercise protocol. We also show good reproducibility of oxidative capacity measured using the short-fast protocol in non-athletic adults.

### Agreement between τ derived after short-fast *versus* long-slow exercise protocols

This study demonstrates, for the first time, comparable measures of oxidative capacity can be derived following two different resistance-band exercise protocols. This finding has important implications for protocol development in both the field of NIRS and ^31^P-MRS, where the reference-standard for PCr recovery can be measured (Šedivý et al., 2015; Menon et al., 2021; Meyerspeer et al., 2020). During ^31^P-MRS, depletion of PCr can be tracked using a long-slow protocol which permits measurements to be made intermittently throughout exercise whilst minimising movement artifact. When conducting NIRS assessments, the recovery of PCr (estimated through repeat measures of oxygen consumption during arterial occlusions) is the measurement of interest, therefore a short-fast protocol, is sufficient to deplete PCr and the potential for movement artifact during exercise is less problematic. Previously, Ryan et al. concluded that NIRS-measured muscle oxidative capacity is comparable across different 15-s exercise performed on a mechanical pedal *versus* electrical stimulation of various randomized contraction frequencies (Ryan et al., 2013b). In line with Ryan et al., this study further demonstrates that NIRS can be used for oxidative capacity assessment using different duration and contraction frequency exercise protocols. A limitation of this study, however, is the use of alternate allocation randomization of exercise protocols. The use of a randomization software would have been a more rigorous approach.

In this study, the mean NIRS-measured oxidative capacity values for both short duration-fast contraction and long duration-slow contraction exercise protocols (35 and 38 s, respectively) are similar to values previously reported (31–35 s) (Nagasawa et al., 2003; Ryan et al., 2013a; Ryan et al., 2014b) and similar to values for PCr recovery previously reported in a similar group of young healthy, non-athletic individuals (McCully et al., 1993; McCully et al., 1994; Forbes et al., 2009; Ryan et al., 2013a; Ryan et al., 2014a).

For future NIRS studies, it is arguably more convenient and time-efficient to employ the 10-s exercise protocol. This is in line with Larsen et al. who propose that a short, moderate-to-vigorous exercise is sufficient to achieve approximately 50% PCr depletion from baseline without inducing acidosis (Larsen et al., 2012). Similar studies comparing oxidative capacity measured across muscle groups or from different positions within the same muscle would permit better characterisation of NIRS reliability.

Several studies have previously employed NIRS to estimate oxidative capacity in individuals with different physical activity levels (Ryan et al., 2013c; Erickson et al., 2013; Lagerwaard et al., 2020), in different age groups (Lagerwaard et al., 2020) and across different muscle groups/locations (Nagasawa et al., 2003; Adami et al., 2020; Lagerwaard et al., 2020). We observed a considerably wide range of time constant values across our YA group for both short-fast and long-slow protocols (10–86 s). We speculate that this may be due to participants undertaking different volumes of physical activity or exercise training, both of which are known to improve skeletal muscle oxidative capacity and therefore reduce τ (McCully et al., 1994). Conversely, detraining or inactivity is known to deteriorate oxidative capacity and lengthen τ (Šedivý et al., 2015). A limitation of our study is that we did not measure habitual physical activity in our participants and therefore, we cannot confirm this speculation. Furthermore, we included a heterogenous population with respect to age, sex and ethnicity into our study which may also contribute to our wide range of oxidative capacity values across individuals (Erickson et al., 2013).

We observed a small bias of ∼3 s shorter τ following the short-fast exercise protocol. One possible explanation for this is that the LS protocol elicited a slightly higher intensity or workload compared to the short-fast protocol, however, the confidence limits around this estimate are wide and therefore the direction of the difference is more likely a chance finding than a systematically shorter TC following the SF protocol. Previous work suggests high intensity exercise severely prolongs the recovery time constants (Arnold et al., 1984; McCully et al., 1993; McCully et al., 1994), likely due to the onset of anaerobic metabolism and associated drop in intracellular pH that impairs the highly active skeletal muscle creatine kinase activity and reduces PCr re-synthesis rate (Arnold et al., 1984; Bendahan et al., 1990). A limitation of NIRS is that it is not possible to measure pH during exercise. To explore this further, we compared the muscle oxygen consumption values measured immediately at the end of exercise (end-exercise musVO_2_). End-exercise musVO_2_ slopes were well-correlated between the two exercise protocols, indicating a similar rate of oxygen consumption at the end of exercise. End-exercise musVO_2_ was on average greater in the short-fast protocol, however, in line with our τ findings, this was also likely a chance finding.

Mono-exponential curves were excluded from our summary analyses based on poor fit (r^2^ < 0.70) and had associated prolonged time constants. It is possible that the prolonged time constants were due to myocellular acidosis leading to a failure of the participant to recover fully within the 3-min window where we monitored recovery, highlighting the importance of monitoring recovery beyond 3 min. However, we cannot rule out a methodological error that may have led to these outliers.

To date, most published studies that assessed muscle oxidative capacity by comparing exercises of different intensities were carried out with ^31^P-MRS. PCr recovery after exercise is independent of exercise intensity as long as pH remains relatively constant, but will be prolonged with decreased pH (McCully et al., 1993; McCully et al., 1994; Walter et al., 1997; Forbes et al., 2009). Forbes et al. demonstrated a similar PCr recovery time constant between low and high-intensity exercise, despite a significantly different end-exercise PCr level, but with similar end-exercise pH. In such a case, fast glycolytic ATP production following higher-intensity exercise offsets the higher post-exercise PCr reduction, thereby resulting in a comparable PCr time constant between lower and higher-intensity exercise (Forbes et al., 2009). Using ^31^P-MRS of different protocols, Walter et al. (1997), Ryan et al. (2013a), and McCully et al. (1993), McCully et al. (1994) documented end-exercise pH of ≥7.00 results in comparable PCr time constants.

Therefore, our study provides novel evidence that, similar to ^31^P-MRS, NIRS can be a reliable alternative for *in vivo* oxidative capacity assessment with different intensity exercise protocols. Compared to the long exercise protocol, the short exercise is arguably more feasible, time efficient and acceptable for participants.

### Reproducibility in non-athletic adults

We present evidence for good agreement between repeated measures of oxidative capacity in the gastrocnemius using the short-fast protocol in a group of non-athletic adults of wide age range (18–86 years old). Our findings are aligned with good reproducibility of these measures in young adults and in lean athletic older adults undertaking NIRS measures from the vastus lateralis muscle (Fennell et al., 2023). NIRS-measured oxidative capacity was also found to be reproducible in OA smokers with and without chronic obstructive pulmonary disease (COPD) (Adami et al., 2018). Interestingly, compared to Fennel et al (Fennell et al., (2023), we obtained a shorter mean time constant despite including obese and older participants. This suggests the variability of oxidative capacity of different muscle, relative to muscle activeness, as the gastrocnemius is the primary locomotor muscle in walking, standing, or in sway (Adami et al., 2018). Another notable strength of this study is that we have included an ethnically diverse population. This is an important contribution to the literature, as previous NIRS studies have predominantly been performed in Caucasian individuals only. In addition, participants in this study were non-athletic and therefore likely reflect the general population. A limitation of this study, however, is that an objective and formal assessment of habitual physical activity levels was not performed. A further strength of our work is that the gastrocnemius muscle was selected as the NIRS measurement site, limiting the NIR light scattering that may occur at other measurement sites where ATT is likely to be much higher. We also utilised an exercise protocol set-up which consisted of a Velcro waistband and plastic resistance band that is MRI compatible, allowing for future applications. Despite selecting the difference signal to account for blood volume shifts and greater signal to noise ratio in our analyses, as has been previously described (Ryan et al., 2012), a general limitation of this work is that we cannot exclude the possibility that this signal might be influenced by changes in local blood volume.

## Conclusion

In conclusion, there is good agreement between different exercise protocols for estimates of skeletal muscle oxidative capacity using NIRS. The short-fast exercise protocol was reproducible in non-athletic adults. Together these findings support NIRS as a valuable non-invasive tool for measuring muscle oxidative capacity in young and older non-athletic adults alike.

## Data Availability

The datasets presented in this article are not readily available because of the sensitive nature of the data collected for this study, but requests to access the dataset from qualified researchers trained in human subject confidentiality protocols may be sent to SJ at the MRC Unit for Lifelong Health and Ageing at UCL. Requests to access the datasets should be directed to siana.jones@ucl.ac.uk.
