# Supplementary material for: Near-infrared spectroscopy (NIRS) in vivo assessment of skeletal muscle oxidative capacity: a comparison of results from short versus long exercise protocols and reproducibility in non-athletic adults
Source: Front Physiol. 2024 Jul 23;15:1429673. doi: 10.3389/fphys.2024.1429673 (PMC11300208; doi:10.3389/fphys.2024.1429673)
Supplement: Supplementary file 1 [file DataSheet1.docx]

**Supplementary Figures**


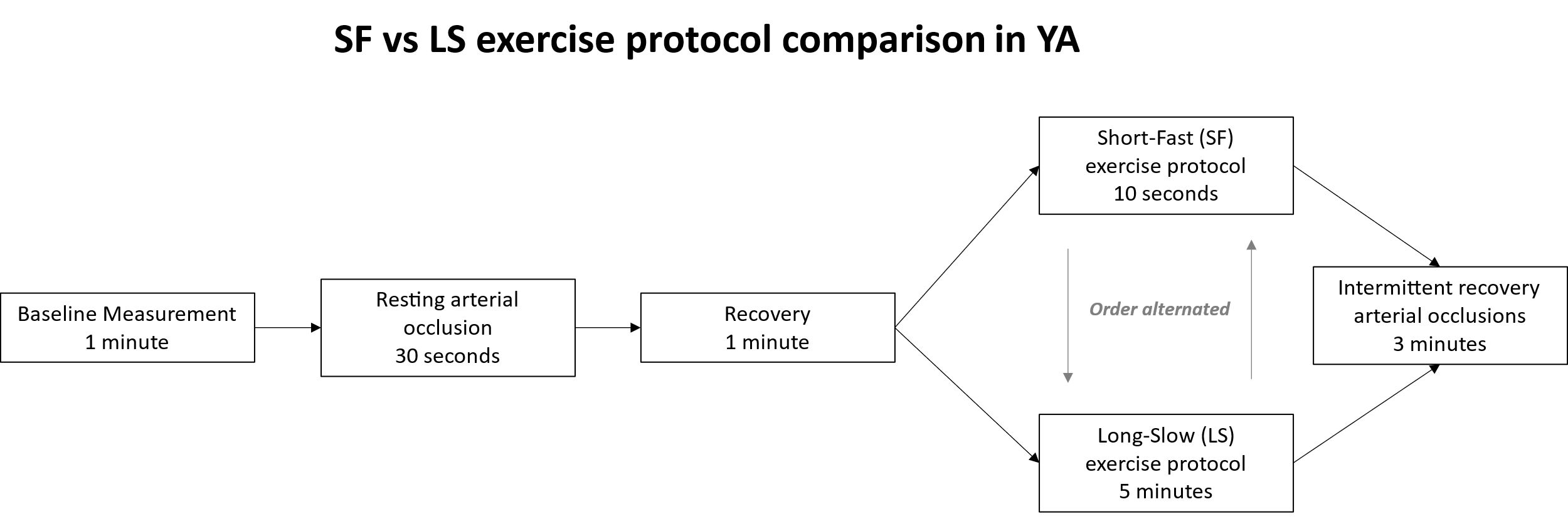


**Supplementary Figure S1.** A schematic representation of the study protocol used in comparing the short-fast and long-slow exercise protocols in young and healthy adult (YA) participants.

**
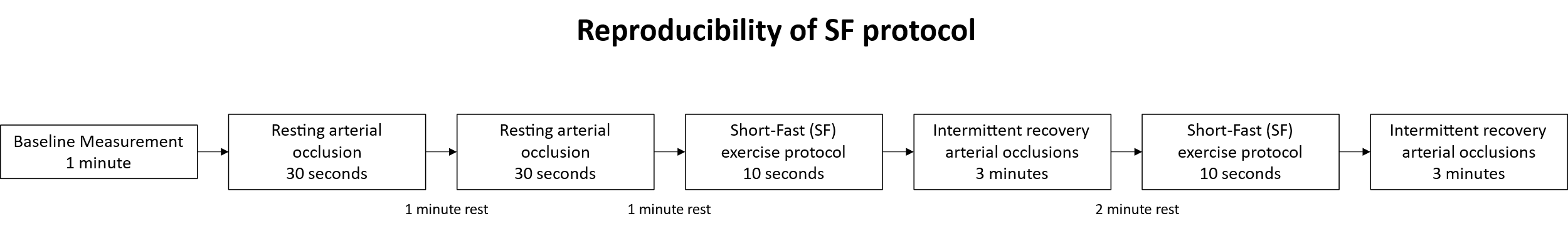
**

**Supplementary Figure S2.** A schematic representation of the study protocol used in the reproducibility study of the short-fast exercise protocol in older adult (OA) individuals.


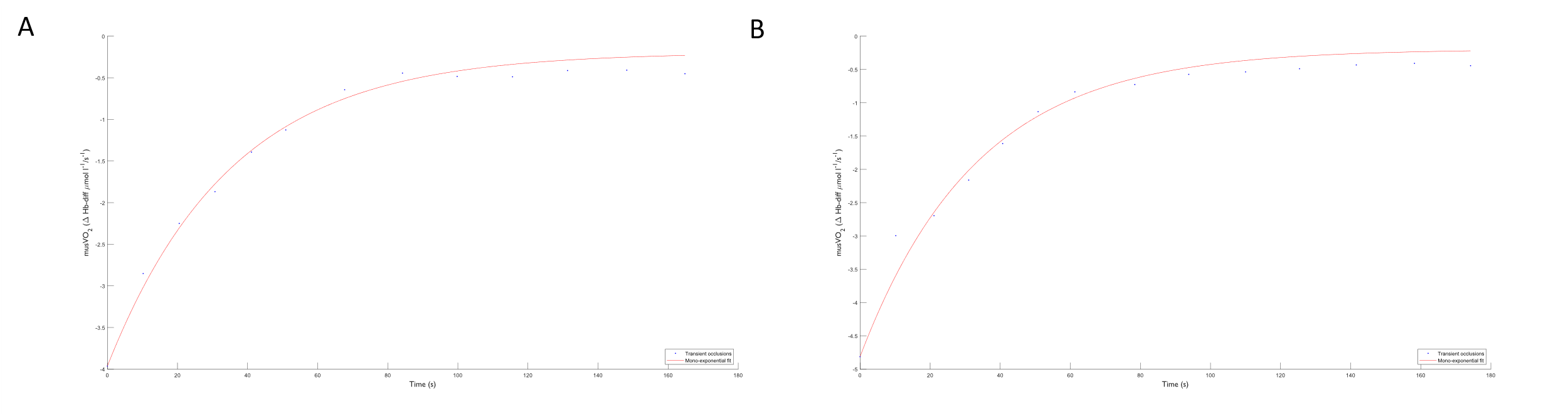


**Supplementary Figure S3.** Representative mono-exponential curves for a young and healthy adult (YA) participant (n=1). (A) Time constant (τ) for the short fast (SF) exercise protocol (τ = 35 seconds) and (B) Time constant (τ) for the long slow (LS) exercise protocol (τ = 33 seconds).

**
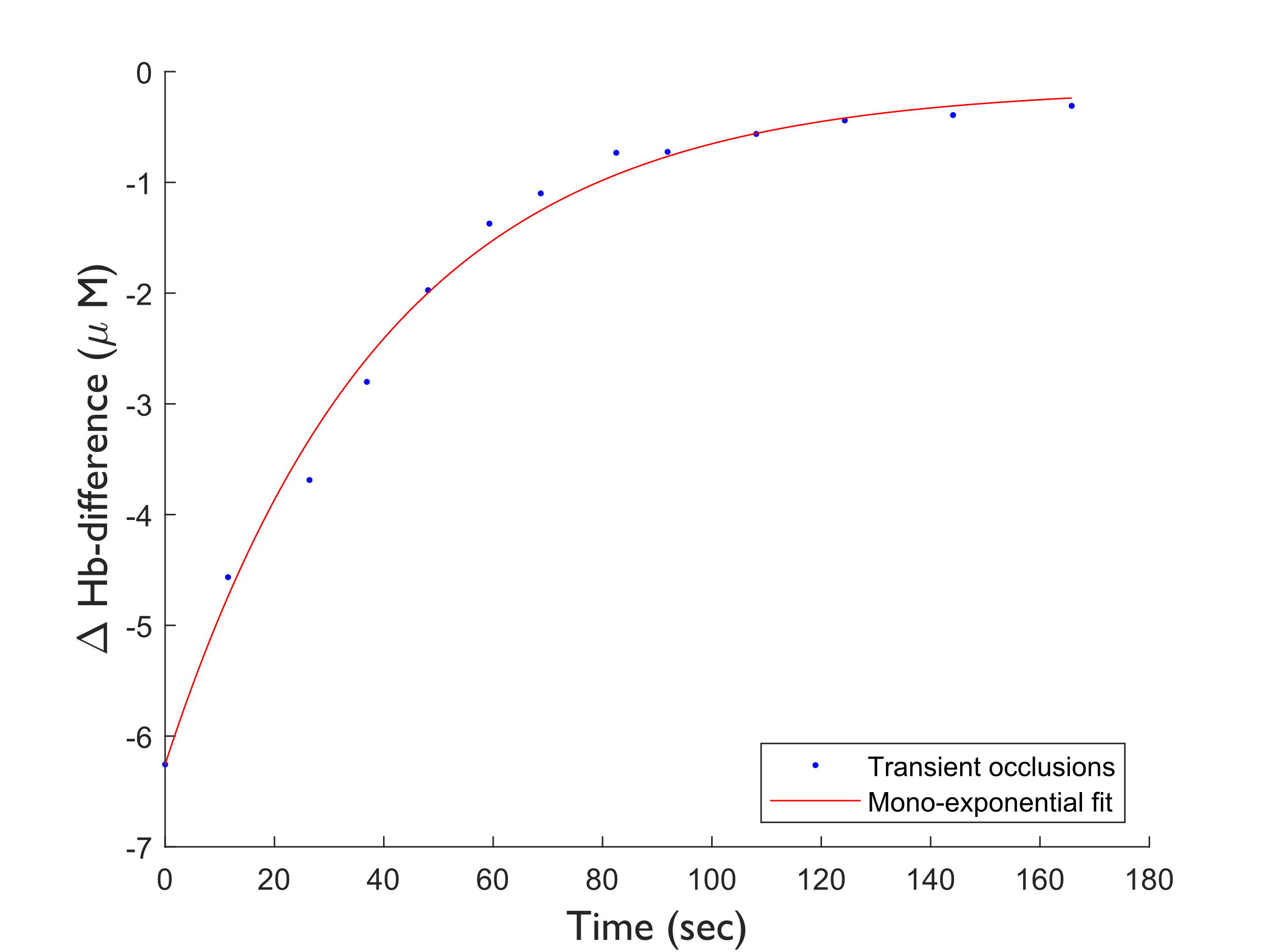
**

**Supplementary Figure S4.** A representative figure of a mono-exponential curve derived for an older adult (OA) participant (n=1). τ = 40.00 seconds.
